# Climate warming decreases the survival of the little auk (*Alle alle*), a high Arctic avian predator

**DOI:** 10.1002/ece3.1160

**Published:** 2014-07-19

**Authors:** Johanna E H Hovinen, Jorg Welcker, Sébastien Descamps, Hallvard Strøm, Kurt Jerstad, Jørgen Berge, Harald Steen

**Affiliations:** 1 Norwegian Polar Institute, Fram CentreTromsø, Norway; 2 University Centre in SvalbardLongyearbyen, Norway; 3 Faculty of Biosciences, Fisheries and Economics, Uit-The Arctic University of NorwayTromsø, Norway; 4 Aurebekksveien 614516, Mandal, Norway

**Keywords:** Adult survival, *Alle alle*, climate change, NAO, SST

## Abstract

Delayed maturity, low fecundity, and high adult survival are traits typical for species with a long-life expectancy. For such species, even a small change in adult survival can strongly affect the population dynamics and viability. We examined the effects of both regional and local climatic variability on adult survival of the little auk, a long-lived and numerous Arctic seabird species. We conducted a mark-resighting study for a period of 8 years (2006-2013) simultaneously at three little auk breeding sites that are influenced by the West Spitsbergen Current, which is the main carrier of warm, Atlantic water into the Arctic. We found that the survival of adult little auks was negatively correlated with both the North Atlantic Oscillation (NAO) index and local summer sea surface temperature (SST), with a time lag of 2 and 1 year, respectively. The effects of NAO and SST were likely mediated through a change in food quality and/or availability: (1) reproduction, growth, and development of Arctic *Calanus* copepods, the main prey of little auks, are negatively influenced by a reduction in sea ice, reduced ice algal production, and an earlier but shorter lasting spring bloom, all of which result from an increased NAO; (2) a high sea surface temperature shortens the reproductive period of Arctic *Calanus*, decreasing the number of eggs produced. A synchronous variation in survival rates at the different colonies indicates that climatic forcing was similar throughout the study area. Our findings suggest that a predicted warmer climate in the Arctic will negatively affect the population dynamics of the little auk, a high Arctic avian predator.

## Introduction

Under stressful conditions, for example during a food shortage, long-lived species are predicted to favor self-maintenance and thus survival, over reproduction (Zera and Harshman 2001). They usually produce a small number of offspring, have a delayed sexual maturity (Lack 1967), and high adult survival rates (Gaillard et al. 1989; Lebreton and Clobert 1991; Sæther et al. 2000;). Thus, even a small alteration in adult survival can have a pronounced influence on population viability (Charlesworth 1980; Gaillard et al. 1989; Wooller et al. 1992; Caswell et al. 1999). Consequently, adult survival is a fitness component that is expected to withstand temporal variability (Sæther and Bakke 2000; Gaillard and Yoccoz 2003). However, this does not exclude the possibility of adult survival being vulnerable to changes in the environment (Gaston and Jones 1998). Climatic variability in particular can strongly affect the normally high survival rate of adults (Durant et al. 2003; Jenouvrier et al. 2003), both directly through changing weather conditions (Schreiber 2002) and indirectly through a change in food quality and/or availability (Sæther et al., 2000; Sandvik et al. 2005).

The effects of global climate change are expected to be strongest in the Arctic. Some of the effects are seen already: glaciers are melting, sea temperatures are rising, and the thickness and extent of sea ice cover are decreasing (IPCC 2013). This can dramatically affect nutrient cycling, food web structure, and species distribution in the Arctic (Prowse et al. 2009; Drinkwater 2011). The major heat source for the Arctic is the warm and saline Atlantic water (Saloranta and Svendsen 2001; Carton et al. 2011). Its temperature and inflow into the Arctic has recently increased and is predicted to increase further in the future (Walczowski and Piechura 2006; Carton et al. 2011; IPCC 2013). The inflow of Atlantic water is influenced by the North Atlantic Oscillation (NAO) (Drinkwater 2011), which is defined as the difference in atmospheric sea level pressure between Stykkisholmur/Reykjavik, Iceland and Lisbon, Portugal. The NAO is the main driving force behind climatic and oceanographic variability in the mid- and high latitudes of the Northern Hemisphere (Hurrell 1995). A positive NAO intensifies the westerlies blowing across the Atlantic, causing a greater inflow of Atlantic water into the Arctic. This leads to higher sea temperatures, less sea ice, reduced ice algal growth, and an earlier onset of phytoplankton bloom in the Arctic. A negative NAO has the opposite effect: weaker winds, reduced inflow of Atlantic water, lower sea temperatures, more sea ice and ice algal production, and later onset of phytoplankton bloom (Visbeck et al. 2001; Arrigo et al. 2008; Drinkwater 2011). Thus, the NAO considerably influences both the weather and oceanographic conditions and thereby also the dynamics and composition of the food webs in the Arctic waters (Drinkwater 2011).

**Figure 1:**
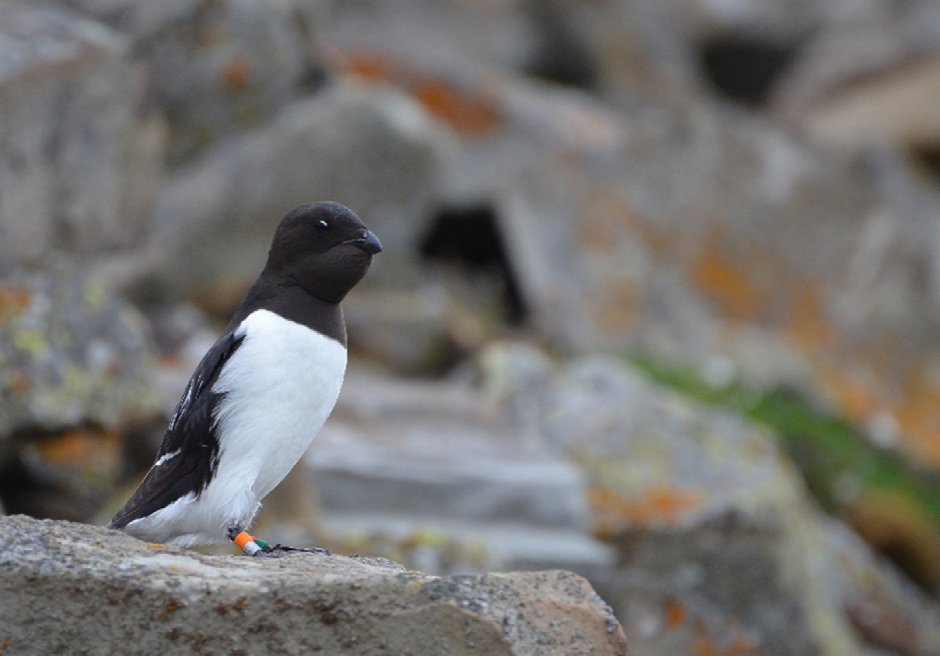
The little auk (*Alle alle*), the most abundant seabird species breeding in the high Arctic. © Benjamin Merkel.

The little auk (*Alle alle*) (Fig. 1) is a long-lived seabird with a single-egg clutch and strong mate fidelity (Stempniewicz 2001; Montevecchi and Stenhouse 2002). It is the most abundant seabird species breeding in the high Arctic (>100 × 10^6^ birds) (Barrett et al. 2006), and based on the information available for closely related alcids (e.g., common murre *Uria aalge* and razorbill *Alca torda*; Friesen et al. 1996), little auks may be expected to live >20 years (Ralph et al. 1995). The little auk has a significant role in the Arctic food web, where it can harvest up to one-fourth of the local standing stocks of zooplankton in a day, depending on the region (e.g., North Water Polynya) (Karnovsky and Hunt 2002). During summer, little auks feed extensively on Calanoid copepods. Preferably those found in cold, Arctic water masses – as these have a higher lipid content, which helps fuel the little auk's increased energy expenditure when rearing the chick (Gabrielsen et al. 1991; Karnovsky et al. 2003; Harding et al. 2009; Kwasniewski et al. 2010; Jakubas et al. 2012). Winter diet is generally assumed to consist of other prey than copepods, such as krill and amphipods (Rosing-Asvid et al. 2013). Arctic copepods are no longer available in winter, as they hibernate at depths below the maximum-diving depth of little auks (∼35 m) (Karnovsky et al. 2011; Brown et al. 2012; Arendt et al. 2013).

In the present study, we tested whether and how climatic variability affects the annual survival rates of adult little auks in the Norwegian Arctic. The low trophic position of little auks in the food web suggests a close link to primary production and thereby makes them well suited for studying the relationship between climate, oceanography, and adult survival of a long-lived marine bird. Furthermore, the little auk has a quite specialized diet (especially during summer, when they eat mainly Arctic calanoid copepods), which implies a high sensitivity to any change in prey abundance and/or availability. As the NAO is known to influence weather, ocean currents, heat transport, and biological productivity in the northern regions (Visbeck et al. 2001; Hurrell et al. 2003; Stenseth et al. 2003; Sandvik and Erikstad 2008; Drinkwater 2011), we used it as a proxy for climatic and, furthermore, feeding conditions for the little auks. In addition to the NAO, we tested whether local sea surface temperature (SST) at the summer breeding grounds of little auks is related to their annual survival rates. In our study area, a strong connection has been found between summer SST and the species composition of local zooplankton communities, with large lipid-rich copepods inhabiting the colder water (Karnovsky et al. 2010). We hypothesized that a positive NAO, as well as a higher SST, would correlate negatively with little auk survival due to increased heat transport, and a reduction in favored, cold water-associated copepods in the Arctic. To test these predictions, we conducted an 8-year mark-resighting study simultaneously at three little auk breeding sites that are influenced by the West Spitsbergen Current (WSC), which is the main carrier of warm, Atlantic water into the Arctic (Saloranta and Svendsen 2001). Given a similar exposure to Atlantic water masses, we expected a parallel response of little auks to climatic variability throughout the study area.

## Materials and Methods

### Study sites

**Figure 2:**
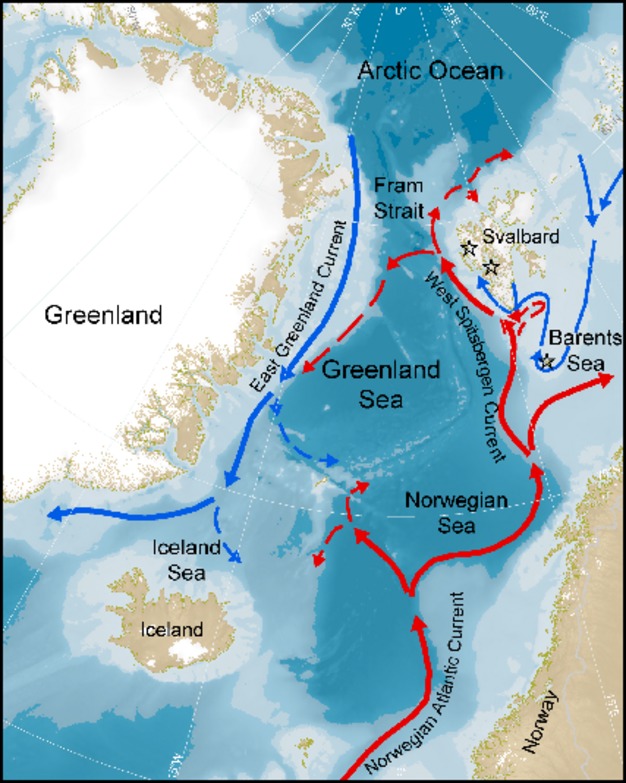
Study area. Red and blue arrows represent the ocean currents transporting warm Atlantic and cold Arctic water masses, respectively. Stars represent the breeding colonies in Bjørnøya (lowermost), Isfjorden (middle), and Kongsfjorden (uppermost star).

We collected data on adult survival of little auks at three breeding sites (Fig. 2): Bjørnøya (74°31′N, 19°01′E), Isfjorden (78°12′N, 15°20′E), and Kongsfjorden (79°01′N, 12°25′E). Bjørnøya is a small island in the western Barents Sea, surrounded by both warm Atlantic and cold Arctic water masses (Weslawski et al. 1999). Isfjorden and Kongsfjorden are open fjords (i.e., they do not have a sill at the mouth of the fjord) located on the western coast of Spitsbergen, Svalbard. Both fjords as well as the adjacent shelf-sea area outside the fjords are regularly supplied with both Atlantic water from the West Spitsbergen Current and Arctic water from the South Cape Current (Hop et al. 2006; Nilsen et al. 2008). Little auks from these breeding sites have been shown to over-winter mainly in the Greenland Sea (Fort et al. 2013).

### Bird captures

We collected mark-resighting data during eight consecutive breeding seasons from 2006 to 2013. Each year, breeding adults were caught with mist nets or noose carpets during late incubation or early chick rearing, and ringed with a stainless steel band and a unique combination of three plastic color leg bands. Also each year, systematic resightings of ringed birds were made. Band reading was spread from mid-June to end of July, and time spent resighting averaged 2 h·day^−1^ for each of the three colonies. During the study, we ringed a total of 1295 birds at the three colonies, of which 220 were never seen again (Table 1). Each year, an average of 128 ± 18 SE birds was resighted (Table 1).

**Table 1:** Little auk (*Alle alle*) mark-resighting data from Bjørnøya (B), Isfjorden (I), and Kongsfjorden (K) 2006–2013. Annual number of identified birds is divided into newly ringed and previously ringed (resighted) individuals. Number of birds seen exclusively in 1 year is also shown.

Year	Colony	Total seen	Newly ringed	Re-sighted	Seen only that year
2006	B	128	128	0	26
	I	106	106	0	22
	K	235	235	0	31
2007	B	192	105	87	18
	I	99	43	56	8
	K	276	88	188	11
2008	B	248	97	151	25
	I	80	11	69	0
	K	233	0	233	0
2009	B	273	76	197	18
	I	98	33	65	1
	K	190	13	177	3
2010	B	290	83	207	11
	I	84	18	66	2
	K	122	4	118	1
2011	B	350	89	261	8
	I	134	65	69	9
	K	136	30	106	5
2012	B	374	62	312	19
	I	134	2	132	0
	K	116	7	109	2
2013	B	272	0	272	0
	I	108	0	108	0
	K	88	0	88	0
Total		4366	1295	3071	220
Average		182	54	128	9
SE		18	12	18	2

### Environmental covariates used to model adult survival

Many studies have shown that the noise ratio of NAO is strongest during the winter months, with far-reaching effects on the dynamics and composition of phyto- and zooplankton communities in the subsequent spring and summer seasons (e.g., Ottersen et al. 2001; Hurrell et al. 2003). Thus, we considered the winter NAO (December through March) index as a proxy for food abundance and availability for little auks during both the breeding and nonbreeding season. In addition, we considered the winter NAO index as an indicator of weather conditions outside the breeding season. We acquired the winter NAO indices from https://climatedataguide.ucar.edu/climate-data.

**Figure 3:**
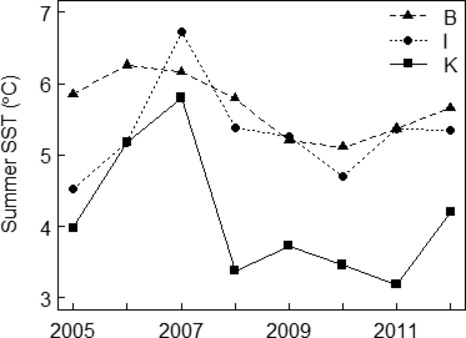
Summer SST (°C) in Bjørnøya (B), Isfjorden (I), and Kongsfjorden (K) during the study period.

In addition to the NAO, we used SST (°C) (Fig. 3) as a proxy for the quality of little auk's foraging ground during summer (Karnovsky et al. 2010). We estimated mean SST for the chick-rearing period (July) of each study year for an area of ∼5500 km^2^ adjacent to each of the Isfjorden and the Kongsfjorden colony, and for an area of ∼23,300 km^2^ adjacent to Bjørnøya (area is larger due to potential foraging grounds at all sides of the island). The areas were chosen such that they covered the maximum-foraging ranges of little auks observed in these areas (∼200 km) (Welcker et al. 2009a; Brown et al. 2012). We acquired SST data at 4 km × 4 km horizontal resolution from the Moderate Resolution Imaging Spectroradiometer (MODIS/aqua; http://disc.sci.gsfc.nasa.gov/giovanni) and computed the July means based on all data points available within the selected areas mentioned above.

Little auks favor old copepodites of *Calanus* copepods, due to their high lipid contents (Scott et al. 2000; Karnovsky et al. 2003; Jakubas et al. 2011). As it takes 2–3 years for cold Arctic water-associated *Calanus* copepods to reach the later stages of their life cycle (Loeng and Drinkwater 2007; Falk-Petersen et al. 2009; Søreide et al. 2010), we considered time lags of up to 3 years in the winter NAO and summer SST. We tested for correlation between the winter NAO and summer SST, and found it to be weak (Pearson's correlation, *r *=* *0.21).

### Adult survival analysis

We used mark-resighting models of the Cormack–Jolly–Seber (CJS) family (Cormack 1964; Jolly 1965; Seber 1965) implemented in the software MARK (White and Burnham 1999) to estimate annual survival rates (*Φ*) while accounting for the potential biases due to variation in resighting probabilities (*P*) (Lebreton et al. 1992). MARK computes the estimates of model parameters *Φ* and p via numerical maximum-likelihood techniques for each survival interval (e.g., from one breeding season to the other) and resighting occasion (e.g., breeding season). We constrained the estimates of *Φ* and *P* between 0 and 1 with the use of a logit-link function. We refer the reader to White and Burnham 1999, and to website http://www.phidot.org/software/mark/ for more details on MARK, its model construction and functions. In model notation, we used the symbols “+” and “×” to refer to additive effects and interactions, respectively. Because of the strong breeding site fidelity of little auks (Norderhaug 1968), we assumed that permanent emigration equals death.

We started the analysis with a fully time- and site-dependent model *Φ*_(COLONY × YEAR)_, *P*_(COLONY × YEAR)_, which allows the survival rate and resighting probability to vary between breeding sites (colonies), and between survival intervals and resighting occasions. We used the program U-CARE to assess its goodness of fit (GOF) to the data (Choquet et al. 2009). In addition to test statistics, GOF tests provide an estimate of the variance inflation factor (ĉ) that quantifies the amount of over-dispersion in the model structure (Burnham and Anderson 2002). The GOF tests comprise four different tests (3.SR, 3.SM, 2.CT, and 2.CL), each testing different aspects of how well the data fits model assumptions – CJS model assumes independence of fates and identity of rates among individuals in the study population. Tests 3.SR and 3.SM test survival independence and identity, whereas tests 2.CT and 2.CL test resighting independence and identity among individuals between breeding seasons (see Lebreton et al. 1992 for a more detailed explanation). The GOF tests rejected the fully time- and colony-dependent model (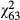 = 279, *P *<* *0.0001) (Table 2). The lack of fit was primarily explained by the 2.CT test (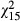 = 180, *P *<* *0.0001, *z *=* *−7.35) (Table 2) indicating a strong effect of trap happiness in the data, that is, individuals were more likely to be resighted if they had been resighted during the previous breeding season (Pradel 1993).

**Table 2:** U-CARE assessed goodness of fit (GOF) of the fully time and colony-dependent model, *Φ*_(COLONY × YEAR)_, *P*_(COLONY × YEAR)_, to the little auk data, where B = Bjørnøya, I = Isfjorden, and K = Kongsfjorden.

Test	Colony	df	χ^2^	P
3.SR	B	6	5.85	0.4407
	I	6	17.31	0.0082
	K	5	1.34	0.9311
3.SM	B	5	6.79	0.2367
	I	7	10.55	0.1594
	K	4	8.88	0.0642
2.CT	B	5	52.80	<0.0001
	I	5	56.44	<0.0001
	K	5	70.71	<0.0001
2.CL	B	4	3.28	0.5122
	I	7	20.00	0.0056
	K	4	25.06	<0.0001
Sum		63	279	<0.0001

To account for trap happiness, we used a multistate approach, based on two states: “seen” and “not seen” (unobservable state). Survival (*Φ*) was set equal for both states, the nominal resighting probability (*P*) was fixed to 1 for the “seen” state and 0 for the “not seen” state, and the actual resighting probability was estimated as the transition probability (*ψ*) to the “seen” state, separately for both states (see Frederiksen et al. 2004a,b2004b). The GOF statistic for this multistate model correcting for trap happiness (*Φ*_(COLONY × YEAR)_, *ψ*_(M × COLONY × YEAR)_, where M = state) was obtained by considering only tests 3.SR, 3.SM, and 2.CL (i.e., excluding the 2.CT test), but there was still evidence for a lack of fit (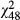 = 99, *P *<* *0.001) (Table 2). To correct for this over-dispersion, we used ĉ = 2.06 (calculated as the *χ*^2^ GOF statistic divided by the degrees of freedom) (Lebreton et al. 1992) in the remaining analyses. Also, as ĉ = 2.06 < 3, the model *Φ*_(COLONY × YEAR)_, *ψ*_(M × COLONY × YEAR)_ was adequate as a general starting model (Lebreton et al. 1992; White and Burnham 1999).

We used the Akaike's information criterion (AIC) in order to select the most parsimonious model(s) (Burnham and Anderson 2002). The AIC penalizes for increasing the number of parameters in the model and thus selects a model that has the best fit with the least parameters. In step one, we addressed whether survival (*Φ*) and resighting (*ψ*) probabilities vary in time and between the colonies by considering all possible models corresponding to a simplification of the general starting model (44 different models). We chose the model(s) with lowest QAIC_c_ (AIC corrected for small sample size and over-dispersion) as good candidate model(s) for step two (Burnham and Anderson 2002). A ΔQAIC_c_ > 2 between the model with lowest QAIC_c_ and a competing model indicates that the competing model has considerably less support. A ΔQAIC_c_ < 2 between the model with lowest QAIC_c_ and a competing model indicates that they cannot be distinguished in their ability to model the data (Burnham and Anderson 2002). When ΔQAIC_c_ < 2 between two nested models, the simplest model was retained. When ΔQAIC_c_ < 2 between two nonnested models, both models were retained. We also considered QAIC_c_ weights to select models for step two (Burnham and Anderson 2002). In step two, we constrained both *Φ* and *ψ* to be linear functions of the winter NAO and summer SST (with and without time lags), while keeping their model structures equal to those of the best models in step one. Like *Φ*, also *ψ* may vary according to interannual variability in environmental conditions. For example, poor-foraging conditions can result in skipped breeding. SST data from the three colonies/areas (Fig. 3) were standardized (*z*-transformed) before their effects on little auk survival were assessed. Additive effects between environmental covariates (NAO + SST, with and without time lags) on *Φ* and *ψ* probabilities were also considered. We used ΔQAIC_c_ and QAIC_c_ weights in a similar manner as in step one for choosing the best final model(s). We report all models with QAIC_c_ weight > 0.01 (after rounding).

## Results

### Model selection step one

**Figure 4:**
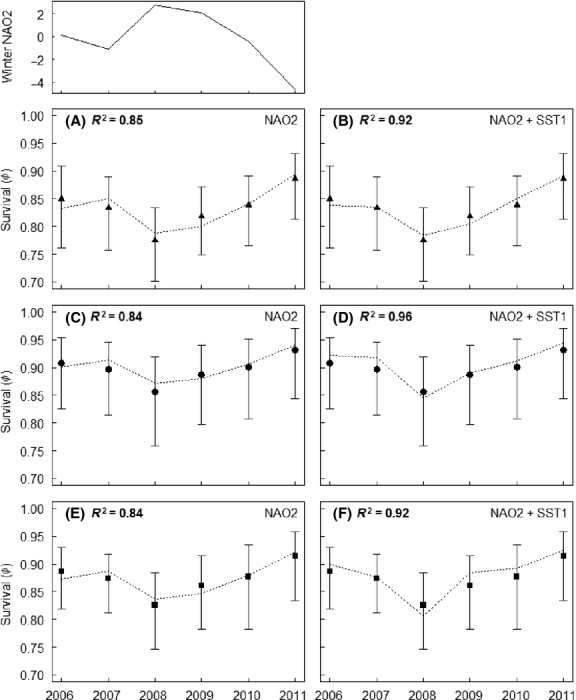
The winter NAO (2-year time lag), and correlation (*R*^2^) between the annual survival rates (± 95% CI) of adult little auks (*Alle alle*) from the model *Φ*_(COLONY + YEAR)_, *ψ*_(M + [COLONY × YEAR])_ (filled symbols) and the predicted annual survival rates from the models *Φ*_(COLONY + NAO2)_, *ψ*_(M + [COLONY × YEAR])_ and *Φ*_(COLONY + NAO2 + SST1)_, *ψ*_(M + [COLONY × YEAR])_ (dashed lines) in (A, B) Bjørnøya, (C, D) Isfjorden, and (E, F) Kongsfjorden.

Resighting probabilities varied both in time and between the colonies (Table 3). Temporal and between-colony variation in survival probabilities was more limited (Table 3). The model with constant survival rate (*Φ*_(.)_) had less support than the models with temporal and/or between-colony variation in survival (Table 3). The model in which survival was forced to vary in parallel in the three colonies (additive colony effect, *Φ*_(COLONY + YEAR)_) had considerably better support than the model in which survival patterns were allowed to differ in the three colonies (interaction between time and colony, *Φ*_(COLONY × YEAR)_) (Table 3). Parallelism suggests that survival varied synchronously at all study sites (Fig. 4).

**Table 3:** Step one and two model selection results. The best models (ΔQAIC_c_ < 2) in both steps are shown in bold. In step two, the year effect in start models (best models from step one) was replaced by the environmental covariates. All models with QAIC_c_ weight >0.01 (after rounding) are shown. Applied over-dispersion factor ĉ = 2.06. *Φ* = survival, *ψ* = resighting probability, M = state (“seen”/“not seen”), NAO = the winter North Atlantic Oscillation (NAO), SST = sea surface temperature (°C). Numbers 1 and 2 after NAO and SST represent time lags of 1 and 2 years, respectively. N_p_ = number of identified parameters, ΔQAIC_c_ = difference between the QAIC_c_ of this model and the QAIC_c_ of the best model, QAIC_c_ weight = the likelihood of this model given the data and the set of alternative models.

Model results	Np	ΔQAIC_c_	QAIC_c_ weight	QDeviance
Step one				
Start model				
*Φ*_(COLONY × YEAR)_ *ψ*_(M × COLONY × YEAR)_	57	27.66	0.000	290.81
Final model(s)				
***Φ***_**(COLONY)**_ ***ψ***_**(M + [COLONY × YEAR])**_	**25**	**0.00**	**0.317**	**328.54**
***Φ***_**(COLONY + YEAR)**_ ***ψ***_**(M + [COLONY** × **YEAR])**_	**30**	**0.31**	**0.271**	**318.71**
***Φ***_**(YEAR)**_ *ψ*_**(M + [COLONY** × **YEAR])**_	**28**	**0.72**	**0.221**	**323.18**
***Φ***_**(.)**_***ψ***_**(M + [COLONY** × **YEAR])**_	**23**	**1.11**	**0.182**	**333.70**
⋮				
*Φ*_(COLONY × YEAR)_ *ψ*_(M + [COLONY × YEAR])_^1^	40	7.28	0.008	305.30
Step two				
Start model(s)				
*Φ*_(COLONY)_ *ψ*_(M + [COLONY × YEAR])_	25	6.47	0.011	328.54
*Φ*_(COLONY + YEAR)_ *ψ*_(M + [COLONY × YEAR])_	30	6.78	0.010	318.71
*Φ*_(YEAR)_ *ψ*_(M + [COLONY × YEAR])_	28	7.19	0.008	323.18
*Φ*_(.)_*ψ*_(M + [COLONY × YEAR])_	23	7.58	0.006	333.70
Final model(s)				
***Φ***_**(COLONY + NAO2)**_ ***ψ***_**(M + [COLONY** × **YEAR])**_	**26**	**0.00**	**0.283**	**320.04**
***Φ***_**(COLONY + NAO2 + SST1)**_ ***vψ***_**(M + [COLONY** × **YEAR])**_	**27**	**0.20**	**0.256**	**318.21**
*Φ*_(COLONY** + **SST1)_ *ψ*_(M + [COLONY × YEAR])_	26	2.08	0.100	322.12
*Φ*_(NAO1 + SST)_ *ψ*_(M + [COLONY × YEAR])_	25	2.58	0.078	324.65
*Φ*_(NAO2)_ *ψ*_(M + [COLONY × YEAR])_	24	2.70	0.073	326.80
*Φ*_(COLONY + NAO1 + SST)_ *ψ*_(M + [COLONY × YEAR])_	27	3.44	0.051	321.45
*Φ*_(NAO2 + SST1)_ *ψ*_(M + [COLONY × YEAR])_	25	4.50	0.030	326.57
*Φ*_(COLONY + NAO)_ *ψ*_(M + [COLONY × YEAR])_	26	5.37	0.019	325.41
*Φ*_(COLONY + NAO1)_ *ψ*_(M + [COLONY × YEAR])_	26	5.54	0.018	325.59
*Φ*_(NAO)_ *ψ*_(M + [COLONY × YEAR])_	24	5.79	0.016	329.88

1 The model allowing survival patterns to differ in the three colonies.

### Model selection step two

Resighting probabilities were not influenced by the environmental covariates considered (the winter NAO and summer SST with or without time lags) – as models including these covariates were not among the selected models (Table 3). Survival probabilities, on the other hand, were influenced by the winter NAO (2-year time lag) (Table 3): the slope *β* (based on a logit-link function) of the linear relation between the winter NAO and survival was -0.11 (95% CI: −0.19 to −0.03), suggesting a negative correlation between survival and the winter NAO (Table S1). The winter NAO affected annual survival rates in a similar way at all breeding sites (additive colony effect, *Φ*_(COLONY + NAO2)_) (Table 3). Strong linear correlations (*R*^2^ = 0.84–0.85) between the predicted survival rates from the model *Φ*_(COLONY + NAO2)_ and survival rates from the model *Φ*_(COLONY + YEAR)_ indicate that temporal variation in survival was mainly explained by the winter NAO with a 2-year time lag (Fig. 4A, C and E).

The second best model (ΔQAIC_c_ < 2) was a model in which survival was negatively correlated with both the winter NAO and summer SST (1-year time lag) (*Φ*_(COLONY + NAO2 + SST1)_) (Table 3, S1). The effect of summer SST on survival was lower than that of the winter NAO: *R*^2^-values increased only by 0.07–0.12 after inclusion of SST (Fig. 4B, D and F), and the slope *β* between the summer SST and survival was −0.24 (95% CI: −0.61 to 0.12) (Table S1). However, also the model *Φ*_(COLONY + SST1)_ had rather strong support (ΔQAIC_c_ = 2.08, which is very close to ΔQAIC_c_ < 2) (Table 3). In this model, the slope *β* between SST and survival was −0.44 (95% CI: −0.80 to −0.08) (Table S1). Models excluding environmental covariates were not well supported with ΔQAIC_c_ > 6 (including the best models from step one) (Table 3).

## Discussion

Our findings clearly show that annual survival rates of adult little auks in the Arctic are influenced by climatic and oceanographic variability: an increase in the winter NAO and summer SST was associated with a decrease in little auk survival rates. Also, the synchronous variation in survival rates between breeding sites indicates that the environmental forcing was similar throughout the study area. The present study is among the first to demonstrate a relationship between regional and local climatic variation and survival of the little auk, a high Arctic avian predator. Only one study has previously investigated the effect of climatic variability on the little auk adult survival, and it did not find any connection between the two (Grémillet et al. 2012). However, the above-mentioned study was performed on a very short timescale (three survival intervals).

The delayed response of little auk survival to both winter NAO and summer SST suggests that their effects were mediated through the food chain. A sufficient amount of high-quality food within foraging distance from the breeding sites (∼200 km for little auks) is a key factor determining body condition and nutritional status of parent seabirds (e.g., Furness and Camphuysen 1997; Sorensen et al. 2008; Kitaysky et al. 2010). Poor body condition and nutritional status during breeding season have previously been shown to negatively affect adult survival of little auks (Welcker et al. 2009b; Harding et al. 2011). The preferred prey of little auks during summer, late life stages of lipid-rich Arctic *Calanus* copepods, vary in abundance depending on primary production. In ice-covered areas, Arctic *Calanus* copepods utilize high-quality ice algae to fuel reproduction during early spring, so that offspring can utilize the phytoplankton bloom 2 months later (Søreide et al. 2010; Leu et al. 2011). In ice-free areas, females may utilize their internal lipid reserves for producing eggs and hence time the growth and development of offspring to the spring bloom (Niehoff and Hirche 2005; Daase et al. 2013). Both these strategies form a copepod stock, which is preyed upon by little auks 2–3 years later – the time it takes for Arctic *Calanus* to complete their life cycle (Falk-Petersen et al. 2009).

Sea ice has a major influence on the Arctic primary production (Smetacek and Nicol 2005): While ice algae need sea ice to grow in, phytoplankton needs ice-free conditions for active photosynthesis. The extent, thickness, and breakup of seasonal ice cover in the Arctic are strongly dependent on sea temperature (Arrigo et al. 2008), which in turn is influenced by the NAO (Ingvaldsen 2005; Drinkwater 2011). A positive NAO increases heat transport into the Arctic (Visbeck et al. 2001). This could negatively influence the Arctic *Calanus* stock size due to less sea ice, implying a reduced ice algal production, and an earlier ice breakup and onset of phytoplankton bloom, which may lead to a mismatch between the ontogenetic development of the *Calanus* offspring and their food supply (Søreide et al. 2010; Leu et al. 2011). Furthermore, in areas where sea ice usually does not form at all, an increase in ocean temperature may initiate an earlier but shorter lasting spring bloom. This could negatively affect the abundance of Arctic *Calanus*, as high food levels to support offspring growth and development would then be available for only a short period of time (Pasternak et al. 2013; Usov et al. 2013). Also, despite their ability to employ a capital breeding strategy, less sea ice, and hence a decreased availability of ice algae, may substantially shorten the reproductive period of Arctic *Calanus* and decrease the number of eggs produced (e.g., Durbin and Casas 2014).

Each of the above-mentioned scenarios would, with a time lag of one to 2 years, result in reduced food availability for little auks in our study area, where both ice-free and ice-covered conditions occur. As a consequence, reduced availability of Arctic *Calanus* may result in an impaired nutritional status of the little auks during breeding season (Harding et al. 2011), with negative effects on the annual survival rates of little auks. The observed effect of NAO on survival rates may also have been mediated through food availability during winter. The main prey of little auks outside the breeding season, krill and amphipods (Rosing-Asvid et al. 2013), may take advantage of sea ice, ice algal blooms, and detrital lumps on the underside of sea ice (Dalpadado et al. 2001; Poltermann 2001; Pinchuk and Hopcroft 2007; Lessard et al. 2010). Furthermore, as their numbers generally correlate negatively with sea temperatures (Coyle et al. 2011), an increased winter NAO may negatively affect both them and little auks preying upon them during winter months.

Little auk survival correlated negatively not only with the NAO, but also with summer SST. High SST may shorten the reproductive period of Arctic *Calanus* females, which are only able to maintain egg production as long as temperature in the upper water layer stays below 5°C (Hirche and Kwasniewski 1997; Niehoff and Hirche 2005). Thus, an increase in summer SST may diminish the local amount of Arctic *Calanus* and hence negatively affect little auks, which depend on energy-rich prey during summer months.

The winter NAO indices ranged from −4.64 up to 2.79 (Fig. 4A), suggesting that the little auks experienced both good and bad weather conditions during the study period (Rivière and Orlanski 2007). However, the absence of a nonlagged effect of the winter NAO in our analyses suggests that weather conditions did not influence the survival rates of little auks outside the breeding season. This may be due to the ability of little auks to perform long migrations (up to 3500 km) (Fort et al. 2013) and thereby to change location if and when weather turns unfavorable. Indeed, direct effects of climate change on seabirds are likely to be less important than indirect effects mediated through the food chain (Sandvik et al. 2005).

Furthermore, annual survival rates varied synchronously throughout the study area. This may indicate that little auks from the three breeding sites experienced similar foraging conditions during summer. All three sites are located in close vicinity of the WSC and are hence strongly influenced by the Atlantic water masses it carries. In addition, little auks from these breeding sites share common wintering grounds (Fort et al. 2013) and may therefore be assumed to experience similar winter foraging conditions. Whether the results of this study are applicable to little auks breeding elsewhere in the Arctic is uncertain: three colonies may not be sufficient to conclude synchronous response of little auks to climatic change across the Arctic. Such a response may, however, be expected in the areas where the environmental conditions and access to foraging grounds are similar.

In general, the annual survival rates of little auks were rather high (on average 0.87, all colonies combined), despite the little auks’ sensitivity to climatic variation. Such a high annual survival rate is to be expected for a long-lived bird with a single-egg clutch (Lack 1968; Gaillard and Yoccoz 2003). However, delayed maturity and low fecundity means that even a small reduction in survival probability can have a strong effect on population dynamics and viability (Gaillard et al. 1989; Sandvik et al. 2012). Sea temperatures and the inflow of Atlantic water are predicted to continue to increase in the Arctic (IPCC 2013), with a reduction in the availability of Arctic zooplankton for little auks as a likely consequence. Our study suggests that this may impair the survival and, hence, decrease the population size of little auks – whose apparently strong breeding site fidelity (Norderhaug 1968; Wojczulanis-Jakubas et al. 2014) likely prevents them to relocate their breeding site when foraging conditions deteriorate. However, also other factors, such as the recruitment of new breeders to a population and natal philopatry (recruitment to natal colony), can strongly affect the seabird population dynamics (e.g., Suryan and Irons 2001; Sandvik et al. 2012). These factors and their potential variation due to climatic change have not yet been investigated in little auks. Hovinen et al. (2014) found that the fledging probability of a little auk chick is negatively correlated with SST, indicating that the little auk populations are suffering not only from reduced adult survival. Further investigations on the actual abundance of food resources available for little auks and their relation to climatic variability are required for strengthening the findings of our study.

## Supporting Information

**Table S1.** Parameter slope (*β*) estimates (±95% CI) for the top three models in step two. *Φ* = survival, NAO2 = the winter NAO (2-year time-lag), SST1 = summer SST (1-year time-lag), Intercept = Isfjorden, K = Kongsfjorden, B = Bjørnøya.
