# Supplementary material for: Climate warming decreases the survival of the little auk (Alle alle), a high Arctic avian predator
Source: Ecol Evol. 2014 Jul 19;4(15):3127–38. doi: 10.1002/ece3.1160 (PMC4161185; doi:10.1002/ece3.1160)
Supplement: Supplementary file 1 — Table S1. Parameter slope (β) estimates (±95% CI) for the top three models in step two. Φ = survival, NAO2 = the winter NAO (2-year time-lag), SST1 = summer SST (1-year time-lag), Intercept = Isfjorden, K = Kongsfjorden, B = Bjørnøya. [file ece30004-3127-sd1.docx]

**Table S1** Parameter slope (β) estimates (± 95% CI) for the top three models in step two. Φ = survival, NAO2 = the winter NAO ( two-year time-lag), SST1 = summer SST (one-year time-lag), Intercept = Isfjorden, K = Kongsfjorden, B = Bjørnøya
